# Utilizing genomics to understand and respond to global climate change

**DOI:** 10.1186/s13059-021-02317-y

**Published:** 2021-03-29

**Authors:** Justin Borevitz

**Affiliations:** Research School of Biology, Australian National University, Canberra, Australia

Last year, as the first wave of the global pandemic hit, many of us re-thought about the larger and accumulating global emergency of climate. How does our life science genomic research play into this? There is a growing body of research showing how species and populations that may have been adapted to their local climate are already or will soon be outside their limits. This will no doubt continue backed by emerging research. But we need to think about going further to understand how to deal with the more variable climates with the extreme weather they bring. This is on top of other global change threats like land clearing, degradation, and invasive species threats. New work must address questions like how will populations adapt? What is the role of standing genetic diversity and intercrossing in building resilient and adaptable populations that are the foundation of resilient ecosystems? This is a key area, where genomics has much to contribute. Furthermore, it is intractable to perform adaptive potential, let alone adaptation studies in each of 100s of species within an ecosystem. How could foundation or keystone species be targeted with studies testing how genetic variation regulates the extended community and ecosystem phenotypes? This could target forest trees, perennial grasses, and crops in the context of more managed agro-ecosystems. What can be gained from wild relatives to enhance adaptation of domesticated organisms? Can other resilient species be more readily domesticated? Assembling new genomes and sequencing populations is only the first step. Associating genetic variants (SNPs, SVs) with local and regional environmental variables across the landscape is an obvious next step, but validating these predictions with new samples and common garden trials is the real critical long term work. These are viable research paths for evolutionary genomics in the 2020s.

Going further, in the global context, wetlands and oceans systems are often neglected and we should encourage papers in this area. Equally challenging will be to explore the huge genomic diversity below our feet, in the soil microbiome. Soils cycle five times more carbon than humans emit in a year and contain a tremendous stock, but little is known about the organisms that process these natural cycles. As (meta)genomics matures, we can begin to dissect this complexity with higher resolution with spatial, temporal, and experimental replication and functional manipulation. Much like transcriptional regulatory networks are becoming common, microbial co-evolutionary networks could be distilled that process dirt into soil and begin to reverse the degradation of once fertile landscapes. Natural genomics experiments are under way in every living system, and new studies will reveal those repeatable evolutionary steps that lead to successful biological innovations.

This ongoing collection of papers in Genome Biology will be a treasure trove of studies attacking big problems with new technological solutions. Some early highlights include temperature and transposons responses in Arabidopsis, thermal and drought selection on standing variation in Drosophila and maize, as well as a prospectus on applications of CRISPR to crops of the future. We welcome further Genome papers from farther afield that deal with climate adaptation, mitigation, and resilience across novel organisms and ecosystems that aim to make direct and/or predictive links to the solutions we urgently need.

